# Mast Cells Modulate Acute Toxoplasmosis in Murine Models

**DOI:** 10.1371/journal.pone.0077327

**Published:** 2013-10-16

**Authors:** Bo Huang, Shiguang Huang, Ying Chen, Huanqin Zheng, Jilong Shen, Zhao-Rong Lun, Yong Wang, Lloyd H. Kasper, Fangli Lu

**Affiliations:** 1 Department of Parasitology, Zhongshan School of Medicine, Sun Yat-sen University, Guangzhou, Guangdong, China; 2 Key Laboratory of Tropical Disease Control (Sun Yat-sen University), Ministry of Education, Guangzhou, Guangdong, China; 3 Department of Periodontology, School of Medicine, Jinan University, Guangzhou, Guangdong, China; 4 The Anhui Provincial Laboratory of Pathogen Biology, Anhui Medical University, Hefei, Anhui, China; 5 Center for Parasitic Organisms, State Key Laboratory of Biocontrol, School of Life Sciences, Sun Yat-sen University, Guangzhou, Guangdong, China; 6 Department of Pathogen Biology, Nanjing Medical University, Nanjing, Jiangsu, China; 7 Department of Microbiology, Immunology, Dartmouth Medical School, Lebanon, New Hampshire, United States of America; Charité, Campus Benjamin Franklin, Germany

## Abstract

The role of mast cells (MCs) in *Toxoplasma gondii* infection is poorly known. Kunming outbred mice were infected intraperitoneally with RH strain *T. gondii*, either treated with compound 48/80 (C48/80, MC activator) or disodium cromoglycate (DSCG, MC inhibitor). Compared with infected controls, infected mice treated with C48/80 exhibited significantly increased inflammation in the liver (*P* < 0.01), spleen (*P* < 0.05), and mesentery (*P* < 0.05) tissues, higher parasite burden in the peritoneal lavage fluids (*P* < 0.01), and increased levels of mRNA transcripts of *T. gondii* tachyzoite surface antigen 1 (SAG1) gene in the spleen and liver tissues (*P* < 0.01), accompanied with significantly increased Th1 cytokine (IFN-γ, IL-12p40, and TNF-α) (*P* < 0.01) and decreased IL-10 (*P* < 0.01) mRNA expressions in the liver, and increased IFN-γ (*P* < 0.01) and IL-12p40 (*P* < 0.01) but decreased TNF-α (*P* < 0.01) and IL-4 (*P* < 0.01) in the spleens of infected mice treated with C48/80 at day 9-10 p.i. Whereas mice treated with DSCG had significantly decreased tissue lesions (*P* < 0.01), lower parasite burden in the peritoneal lavage fluids (*P* < 0.01) and decreased SAG1 expressions in the spleen and liver tissues (*P* < 0.01), accompanied with significantly increased IFN-γ (*P* < 0.01) and IL-12p40 (*P* < 0.05) in the liver, and decreased IFN-γ (*P* < 0.05) and TNF-α (*P* < 0.01) in the spleens; IL-4 and IL-10 expressions in both the spleen and liver were significantly increased (*P* < 0.01) in the infected mice treated with DSCG. These findings suggest that mediators associated with the MC activation may play an important role in modulating acute inflammatory pathogenesis and parasite clearance during *T. gondii* infection in this strain of mice. Thus, MC activation/inhibition mechanisms are potential novel targets for the prevention and control of *T. gondii* infection.

## Introduction

*Toxoplasma gondii* is a common and significant obligate intracellular pathogen of humans and animals, which infects nearly one third of the human population and is found in an extraordinary range of vertebrate hosts [1]. The immune/inflammatory response to *T. gondii* infection is essential to control parasite replication and tissue spread but also can cause tissue damage, being decisive to pathogenesis [2]. Mast cells (MCs) are abundant in tissues exposed to the external environment including the skin, intestinal tract, and trachea, and also normally present in heart, lymph nodes, spleen, and CNS [3]. Classically, MCs have been described as essential effector cells of immediate hypersensitivity and chronic allergic reactions that contribute to asthma, atopic dermatitis, and other allergic diseases [4]. Recent findings indicate that MCs are more functionally diverse than previously described. MCs play a critical role in host defense against invading microbes and participate in allergy as well as host defence against helminth parasites [5,6]. Additionally, MCs play a role in many different processes, and they are involved in responses against pathogenic infections and most notably in connection with innate immune responses, wound healing, and inflammatory disease [7]. Recent studies indicate that MCs are also involved in immune regulation [8,9]. 

MCs are recognized as effector cells involved in clearance of diverse parasites, including *Trichinella spiralis* and *Trichomonas vaginalis* [10,11]. MC deﬁcient mice infected with *Leishmania major* develop larger lesion load with increased parasitemia [12]. Although MCs are involved during the acute phase of the inflammatory response to *T. gondii* infection in animal model [13], the mechanism by which MC alter the immune response during *T. gondii* infection has not been resolved. In the present study, we assessed the role of MC during acute murine *T. gondii* infection; our findings suggest that release of mediators after MC activation plays an important role in modulating inflammatory pathogenesis and parasite load during acute *T. gondii* infection.

## Materials and Methods

### Ethics Statement

Female 6-week-old Kunming (KM, outbred) mice were obtained from the Animal Center of Sun Yat-sen University, maintained in specific-pathogen-free environment, and had free access to a commercial basal diet and tap water *ad libtum*. Animals were provided with humane care and healthful conditions during their stay in the facility. All individuals who use animals received instruction in experimental methods and in the care, maintenance, and handling of mice; and all efforts were made to minimize animal suffering. Animals were sacrificed using CO_2_ asphyxiation and the appropriate organs were harvested. The protocol in this study was approved by the Committee on the Ethics of Animal Experiments of the Sun Yat-sen University [Permit Numbers: SCXK (Guangdong) 2009–0011]. 

### Parasite

*T. gondii* RH strain tachyzoites were propagated by intraperitoneal (i.p.) passage in KM mice at 4 or 5 day intervals. Mice were infected with 1×10^2^ RH strain *T. gondii* tachyzoites by i.p. injection, and tachyzoites were enumerated using manual counting with a haemocytometer. 

### Mast cell (MC) activation and stabilization in vivo

Total 48 KM mice were included in this study. Mice were divided into 6 groups, consisting of 7-9 mice per group. Compound 48/80 (C48/80) activated the MCs and disodium cromoglycate (DSCG) stabilized the MCs in mice. The model of MC degranulation or stabilization used in the present study was based on a well-characterized protocol with modiﬁcations [14]. Briefly, mice received the first i.p. injection of C48/80 (Sigma-Aldrich, 4 mg/kg/d) or DSCG (Sigma-Aldrich, 25 mg/kg/d) 24 h before infection with *T. gondii* RH strain tachyzoites, and each animal received daily i.p. injection for the duration of the experiment thereafter [9-10 days post infection (p.i.)]. C48/80 enhanced MCs releasing their mediators and DSCG prevented MCs from releasing their mediators for the duration of the experiment. Infected control mice were infected with *T. gondii* RH strain tachyzoites alone without any treatment. Uninfected controls were received injections with either the phosphate-buffered saline (PBS) diluent, C48/80, or DSCG. 

### Histopathological analysis

Mice were sacrificed by CO_2_ asphyxiation prior to death after infection, and their livers, spleens, and mesenteries were harvested and immediately fixed in 10% buffered natural formaldehyde (Guangzhou chemical reagent factory, China). Four-micrometer-thick sections (50- or 100-μm distance between sections) of the organ from each mouse, stained with hematoxylin and eosin (H&E) (Sigma-Aldrich), were evaluated for histological changes. Blinded samples were submitted for semi-quantitative histopathologic analysis. The histological scores in the spleen and mesentery tissues were determined under ×100 magnification in three non-contiguous sections from four mice in each group. In brief, the score used to measure the intensity of spleen and mesentery tissue alterations was 1, 2, 3, and 4 (absent, mild, moderate, and severe, respectively) [15]. Liver sections were analyzed for the numbers of inflammatory foci according to previous report with minor modiﬁcations [2], and the number of inflammatory foci per field was analyzed at a magnification of ×100 under a light microscopy by counting 10 fields of each section at 9-10 days p.i. in each group. All the analyses were performed by two researchers.

### Toluidine blue staining for MCs

Serialized 4-μm-thick sections of spleen and mesentery were deparaffinized, rehydrated, and stained with 0.5% toluidine blue (Sigma-Aldrich) for 120 min. MCs, in three to five sections per animal on days 9 to 10 after treatment, were identified by their deep blue-purple staining and counted at ×400 magnification under light microscopy. MC count was expressed as the number of positive cells *per* mm^2^ and the results were expressed as the mean value of MCs *per* group. MC degranulation was determined as a loss of MC membrane integrity with extrusion of intracellular granules to the extracellular space or MCs completely lacking in intracellular granules as described previously [16]. Completely degranulated MCs with absence of the cytoplasmic granules are invisible by toluidine blue staining. 

### Immunofluorescence staining of tryptase for MCs

  Spleen and mesentery tissue sections (4-μm) were deparaffinized and rehydrated in distilled water. Heat-induced antigen retrieval was carried out in an 800-W microwave oven for 30 min. Endogenous peroxidase activity was blocked by incubation with 0.3 % hydrogen peroxide in methanol for 10 min at room temperature. Non-specific binding was blocked by incubation in PBS containing 10 % normal goat serum and 1 % bovine serum albumin (BSA) (pH 7.4) for 60 min at room temperature. Sections were incubated with anti-MC tryptase mouse monoclonal antibody (AA1, IgG1; 1 mg/ml, 1:200 dilution; Abcam, USA) overnight at 4°C. Slides were then rinsed three times with PBS (pH 7.4) and exposed to secondary antibody [anti-mouse IgG (H+L), F (ab') _2_ fragment (Alexa Fluor^®^ 488 Conjugate); 2 mg/ml, 1:200 dilution; CST, USA] for 60 min at room temperature in a dark chamber. The slides were washed three times with PBS (pH 7.4) for 30 min at room temperature and mounted by antifade polyvinylpyrrolidone mounting medium (Beyotime, China) in a dark chamber. MCs were identified by their green fluorescence staining and counted at ×400 magnifications under a light microscope. Positively stained MCs were counted and expressed as mentioned above.

### *T. gondii* tachyzoite burden in mouse peritoneal lavage fluids

To examine the effect of C48/80 or DSCG on the parasite proliferation in vivo, we examined parasite burden in mouse peritoneal lavage fluids infected with *T. gondii* with either C48/80 or DSCG treatment, or without treatment. Mice were killed at 9-10 days p.i. prior to death after infection, the peritoneal lavage fluids of each mouse was passed through a 27 gauge needle, and the parasite numbers were counted by hemocytometer.

### Measurement of mRNA expression in spleen and liver tissues using quantitative real-time PCR (qRT-PCR)

Total RNA was extracted from about 100 mg spleen or liver sample each mouse using RNA extraction kit (TaKaRa, Japan) according to the manufacturer’s protocol. The quality of total RNA was analyzed by running 5 μl of each RNA sample on a 1.0% agarose gel and visualizing with ethidium bromide. The quantity of total RNA was estimated by measuring the absorbance at 260 nm and 280 nm using a NanoDrop 2000 spectrophotometer (NanoDrop Technologies). First-strand cDNA was constructed from 1.0 μg of total RNA with oligo (dT) as primers using PrimeScript 1^st^ Strand cDNA Synthesis Kit (TaKaRa), following the manufacturer’s protocol. cDNA was stored at −80 °C until use. 

To determine the levels of mRNA transcripts of *T. gondii* tachyzoite surface antigen 1 (SAG1) gene and cytokines including IFN-γ, TNF-α, IL-4, IL-10, and IL-12p40 in both spleen and liver tissues from different groups of mice, qRT-PCR was performed using SYBR Green qPCR Master Mix (TaKaRa) according to manufacturer’s instructions. Primers are listed in Table 1. Brieﬂy, the total 10 μl reaction mixture contained 5.0 μl of SYBR^®^ Premix Ex Taq^TM^ (2×), 0.5 μl of each primer (10 pM), 3.0 μl of dH_2_O, and 1.0 μl of cDNA (0.2 μg/μl). The thermal cycling conditions consisted of an initial denaturation of 30 sec at 95°C followed by 43 cycles of 95°C for 5 sec and 60°C for 20 sec. Values are means from triplicate measurements, speciﬁc mRNA expression levels were normalized to the housekeeping gene β-actin mRNA and the results are expressed as the fold change compared to uninfected controls.

**Table 1:** Primer sequences of mouse target cytokines and housekeeping genes used for quantitative real-time polymerase chain reaction (qRT-PCR) assays.

**Genes**	**Primer sequence (5′→3′)**		**References**
IFN-γ	Forward primer	GGAACTGGCAAAAGGATGGTGAC	[42]
	Reverse primer	GCTGGACCTGTGGGTTGTTGAC	
TNF-α	Forward primer	CCCTCACACTCAGATCATCTTCT	[43]
	Reverse primer	GCTACGACGTGGGCTACAG	
IL-4	Forward primer	ACAGGAGAAGGGACGCCAT	[44]
	Reverse primer	GAAGCCCTACAGACGAGCTCA	
IL-10	Forward primer	AGCCGGGAAGACAATAACTG	[42]
	Reverse primer	CATTTCCGATAAGGCTTGG	
IL-12p40	Forward primer	CCTGGTTTGCCATCGTTTTG	[42]
	Reverse primer	TCAGAGTCTCGCCTCCTTTGTG	
SAG1	Forward primer	CTGTCAAGTTGTCTGCGGAAGGAC	[42]
	Reverse primer	CGTTAGCGTGGCACCATTATCACTC	
β-actin	Forward primer	TGGAATCCTGTGGCATCCATGAAAC	[42]
	Reverse primer	TAAAACGCAGCTCAGTAACAGTCCG	

### Statistical Analysis

Data are expressed as means ± SEM. All of the pathological measurements were done in a blind fashion, and the quantitative measurements were made twice. A statistical software program SPSS 17.0 was applied for analysis. Differences of histopathological examination in liver, spleen, and mesentery between different groups were investigated using the Kruskal-Wallis rank sum test. The fold changes of SAG1 and cytokine mRNA expressions were analyzed by Student's *t* test. A *P*-value of < 0.05 was considered statistically significant. 

## Results

### Survival of mice

**Figure 1:**
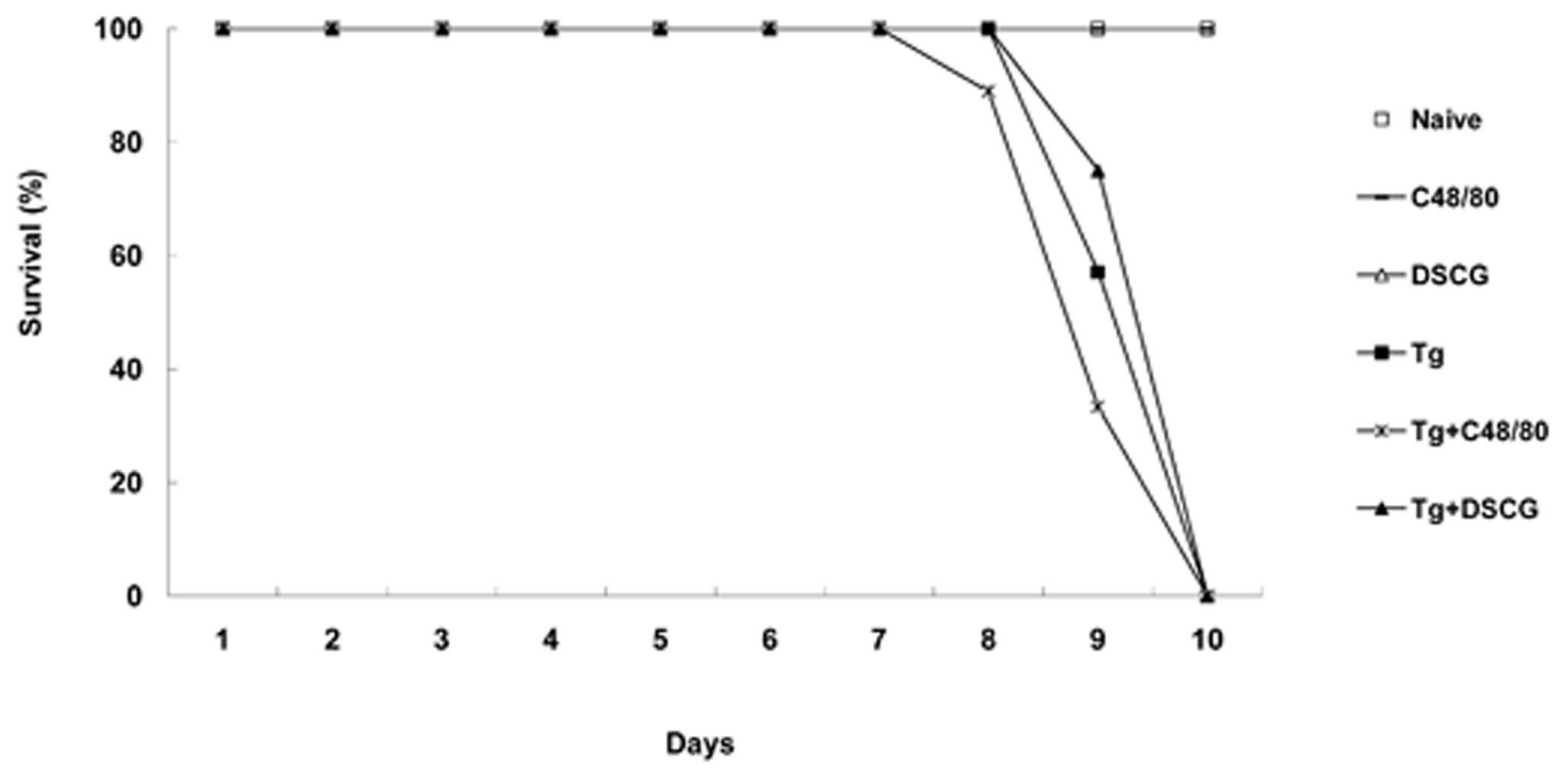
Mice survival after infection with 10^2^ RH tachyzoites of *T*. ***gondii***. Survival of naïve mice treated with PBS (open square, n=8); uninfected mice treated with C48/80 (dash, n=8); uninfected mice treated with DSCG (open upright triangle, n=8); *T. gondii*-infected control mice (filled square, n=7), *T. gondii*-infected mice with C48/80 treatment (asterisk, n=9), and *T. gondii*-infected mice with DSCG treatment (filled upright triangle, n=8). The mice were monitored for survival on a daily basis until the termination of the experiment.

The survival rates and survival times of the infected mice from different groups were similar, and all the RH strain *T. gondii*-infected mice with either C48/80 or DSCG treatment, or without treatment died within 9-10 days p.i. (Figure 1). 

### MC activation and stabilization

**Figure 2:**
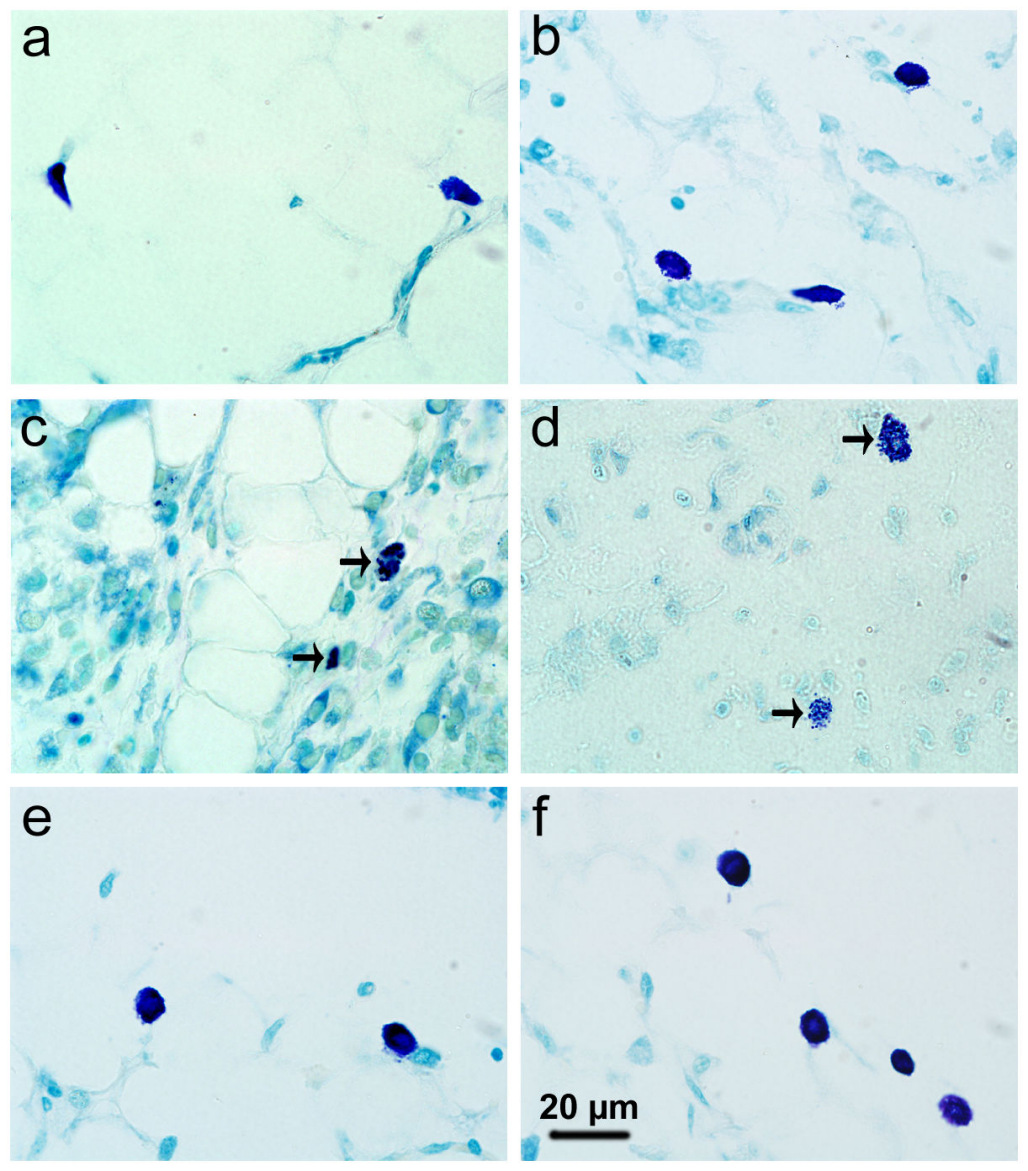
Light photomicrographs of metachromatic MCs in mesenteries by toluidine blue staining. Infected mice i.p. inoculated with 10^2^ RH tachyzoites of *T. gondii* from different groups were killed at 9-10 days p.i. Metachromatic MCs were evaluated in mesentery tissue from uninfected mouse treated with PBS (a), infected control mouse displaying mildly degranulated MCs (b), uninfected mouse treated with C48/80 (c) and infected mouse treated with C48/80 (d), both displaying degranulated MCs (arrows); uninfected mouse treated with DSCG (e) and infected mouse treated with DSCG (f), both displaying intact MCs.

**Figure 3:**
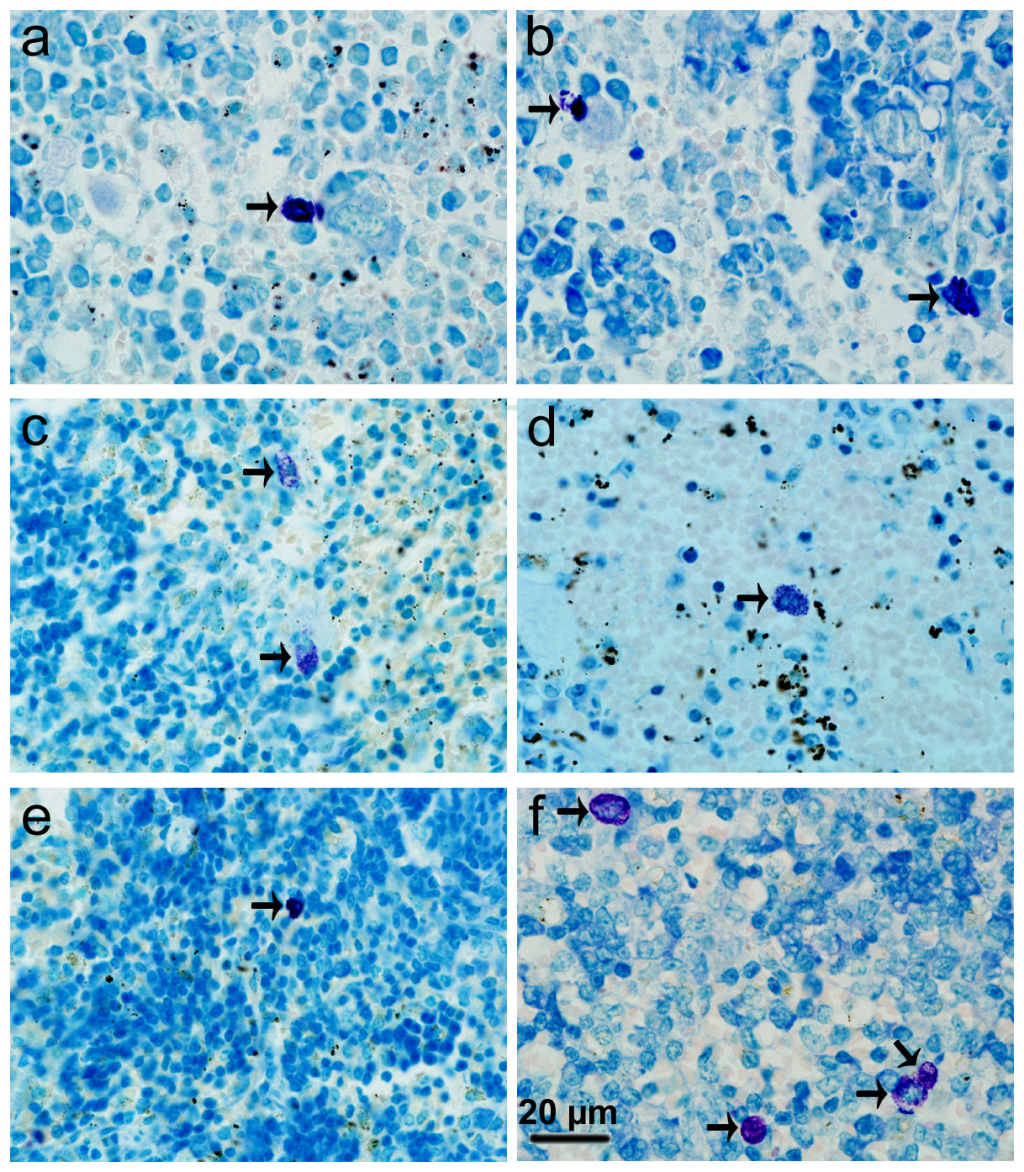
Light photomicrographs of metachromatic MCs in spleens by toluidine blue staining. Infected mice i.p. inoculated with 10^2^ RH tachyzoites of *T. gondii* from different groups were killed at 9-10 days p.i. Metachromatic MCs (arrows) were evaluated in spleen tissue from uninfected mouse treated with PBS (a), infected control mouse displaying a degranulated MC (b), uninfected mouse treated with C48/80 (c) and infected mouse treated with C48/80 (d), both displaying degranulated MCs; uninfected mouse treated with DSCG (e) and infected mouse treated with DSCG, both displaying intact MCs (f).

**Figure 4:**
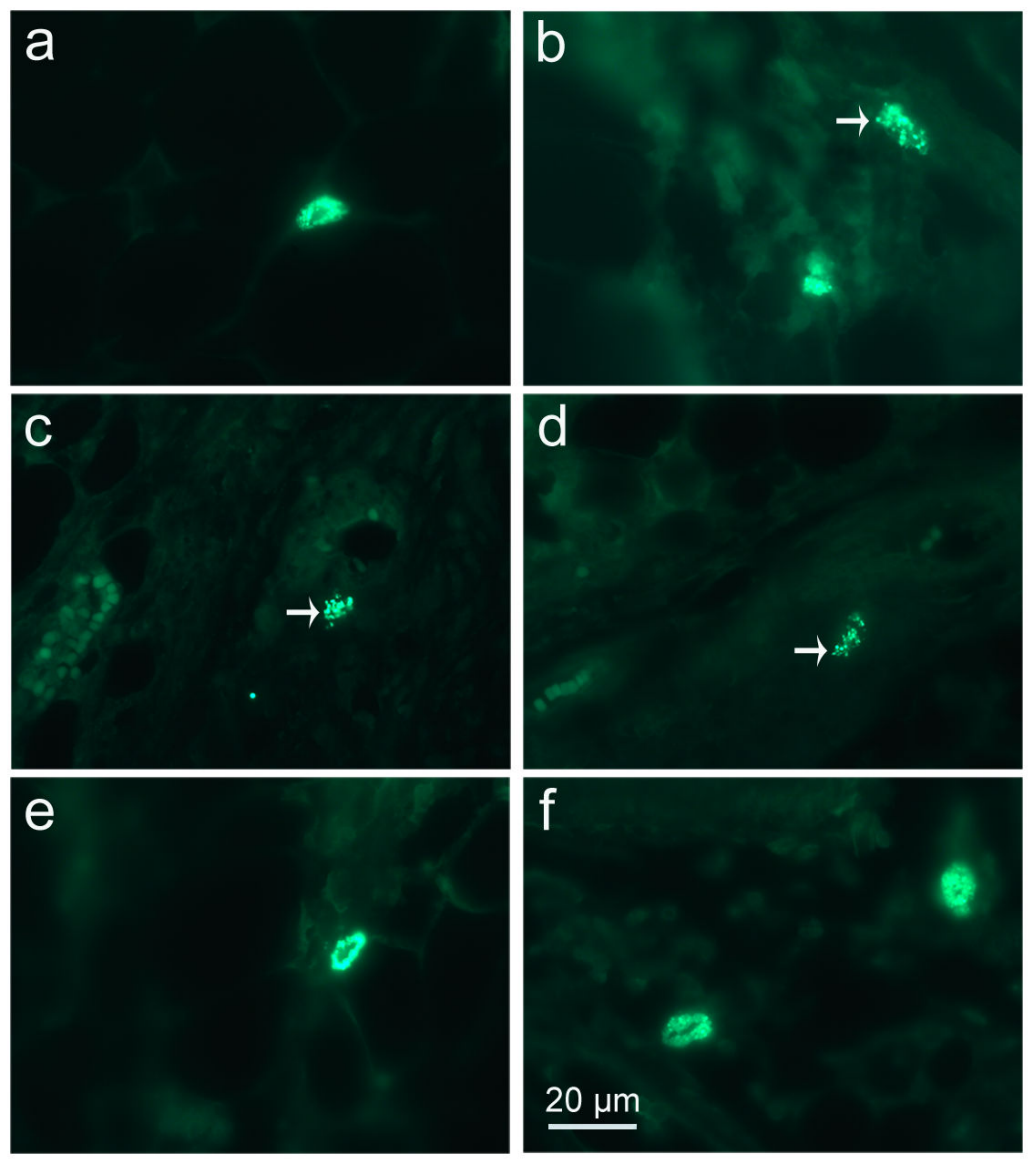
Light photomicrographs of tryptase positive-MCs in mesenteries by immunofluorescence staining. Infected mice i.p. inoculated with 10^2^ RH tachyzoites of *T. gondii* from different groups were killed at 9-10 days p.i. MCs were evaluated in mesentery tissue from uninfected mouse treated with PBS (a), infected control mouse displaying a degranulated MC (arrow) (b), uninfected mouse treated with C48/80 (c) and infected mouse treated with C48/80 (d), both displaying degranulated MCs (arrows); uninfected mouse treated with DSCG (e) and infected mouse treated with DSCG (f), both displaying intact MCs.

**Figure 5:**
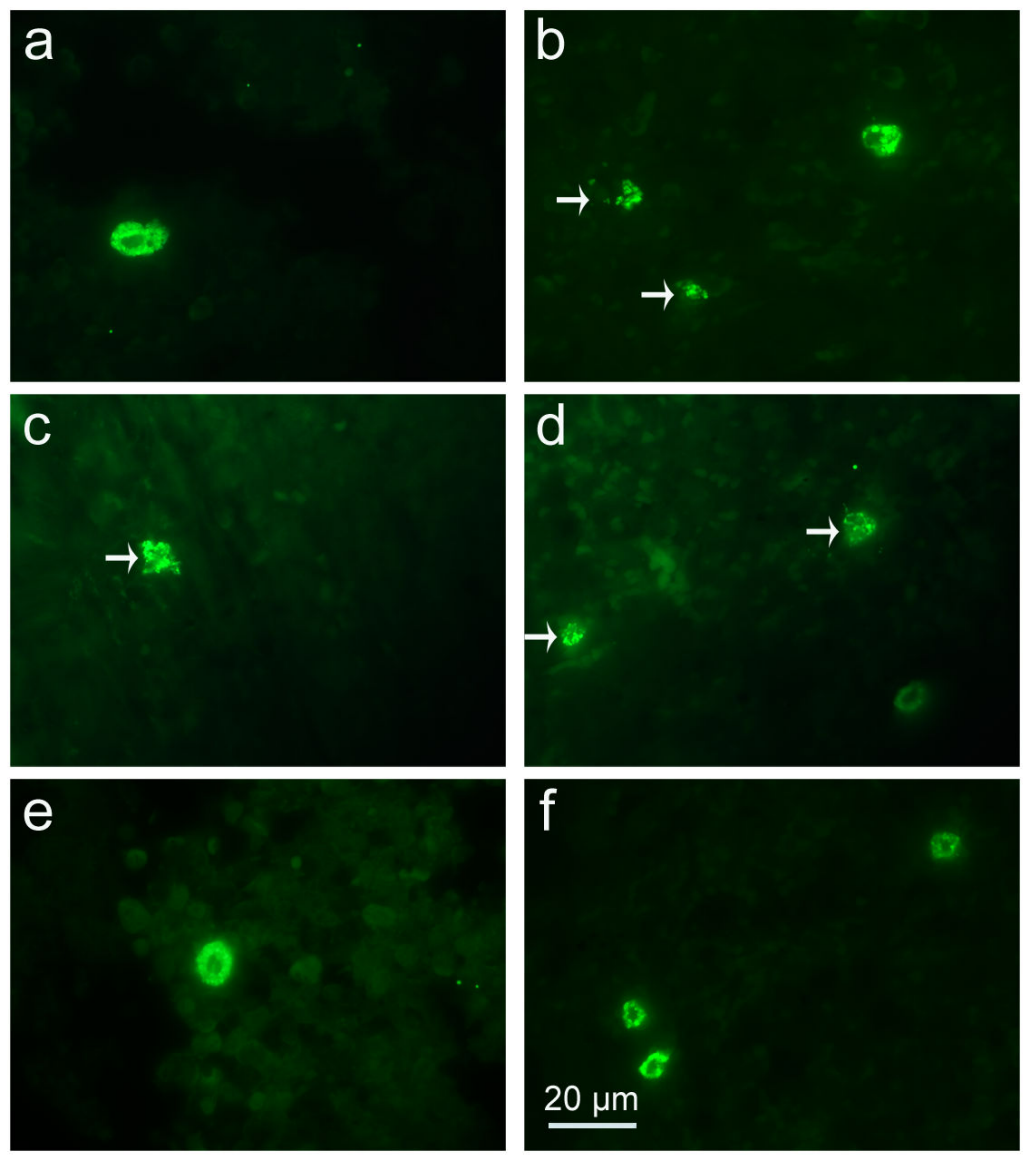
Light photomicrographs of tryptase positive-MCs in spleens by immunofluorescence staining. Infected mice i.p. inoculated with 10^2^ RH tachyzoites of *T. gondii* from different groups were killed at 9-10 days p.i. MCs were evaluated in spleen tissue from uninfected mouse treated with PBS (a), infected control mouse displaying degranulated MCs (arrows) (b), uninfected mouse treated with C48/80 (c) and infected mouse treated with C48/80 (d), both displaying degranulated MCs (arrows); uninfected mouse treated with DSCG (e) and infected mouse treated with DSCG (f), both displaying intact MCs.

Stained with toluidine blue, MCs were identified in tissue sections from their characteristic granular, deep blue-purple metachromatic appearance against blue orthochromatic background tissue. Toluidine blue stained sections of the mesenteries and spleens from different groups at 9-10 days p.i. were shown in Figures 2 and 3, respectively. Stained with immunofluorescence for tryptase, MCs from their characteristic green fluorescence were identified in tissue sections of the mesenteries and spleens from different groups at 9-10 days p.i. (Figures 4 and 5, respectively). MCs were intact in uninfected mice with PBS treatment (Figures 2a, 3a, 4a, and 5a); MCs had mild or obvious granula release (Figures 2b, 3b, 4b, and 5b) in *T. gondii*-infected control mice. However, MCs had marked granule release in uninfected (Figures 2c, 3c, 4c, and 5c) and *T. gondii*-infected mice (Figures 2d, 3d, 4d, and 5d) with C48/80 treatment. MCs were intact in uninfected (Figures 2e, 3e, 4e, and 5e) and *T. gondii*-infected mice (Figures 2f, 3f, 4f, and 5f) with DSCG treatment, and the latter appeared morphologically indistinguishable from the uninfected controls. 

### Spleen MC densities

**Figure 6:**
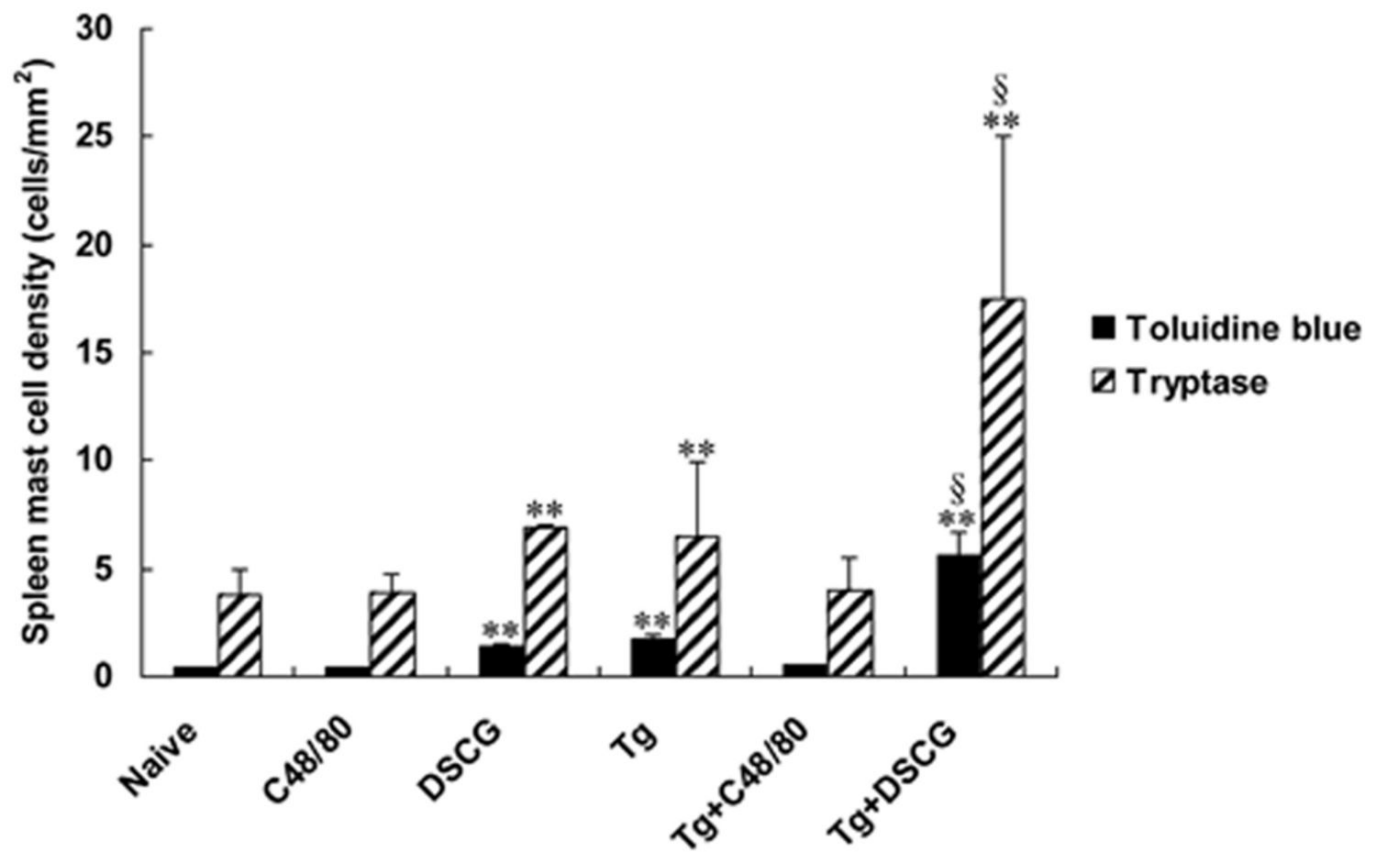
The numbers of metachromatic and tryptase-positive MCs in spleen tissues from different groups expressed as MCs mm^−2^. There were 4 mice per group, and the data are representative of two experiments. Statistically significant differences for comparison with the uninfected mice with PBS (**, *P* < 0.01) and for comparison with the infected controls (§, *P* < 0.01).

MC count was assessed by examining sections of spleen tissues by both metachromatic staining with toluidine blue and immunofluorescence staining of tryptase. As shown in Figure 6, there were only a low density (the number of MCs *per* mm^2^) positively stained MCs with undegranulation observed in the spleen tissues of uninfected mice treated with PBS, while there were significantly higher densities of MCs in *T. gondii*-infected control mice. In uninfected mice, C48/80 administration did not change the number of MCs; while DSCG administration increased the MC density in the spleens by 3.1 fold by toluidine blue staining (*P* < 0.01) and 1.8 fold by immunofluorescence staining of tryptase (*P* < 0.01) relative to that in uninfected mice with PBS. *T. gondii* infection increased the density of MCs by 4.0 fold by toluidine blue staining (*P* < 0.01) and 1.7 fold by immunofluorescence staining of tryptase (*P* < 0.01) relative to that in uninfected mice with PBS. In contrast, in *T. gondii*-infected mice that received C48/80, the density of MCs was no change by both staining, whereas in *T. gondii*-infected mice that received DSCG, the density of MCs was increased by 13.0 fold by toluidine blue staining (*P* < 0.01) and 4.6 fold by immunofluorescence staining of tryptase (*P* < 0.01) relative to that in uninfected mice with PBS. Compared with toluidine blue staining, there were significantly higher MC densities in spleen tissues in all the groups when using immunofluorescence staining of tryptase (*P* < 0.01). C48/80 treatment of the spleens degranulated MCs, which resulted in a lack of both toluidine blue staining of granule matrix proteoglycans and immunofluorescence staining of tryptase. However, it is important to notice that not all MCs were degranulated or undegranulated by these treatments.

### Severe liver, spleen, and mesentery inflammation in *T. gondii*-infected mice with C48/80 treatment

**Figure 7:**
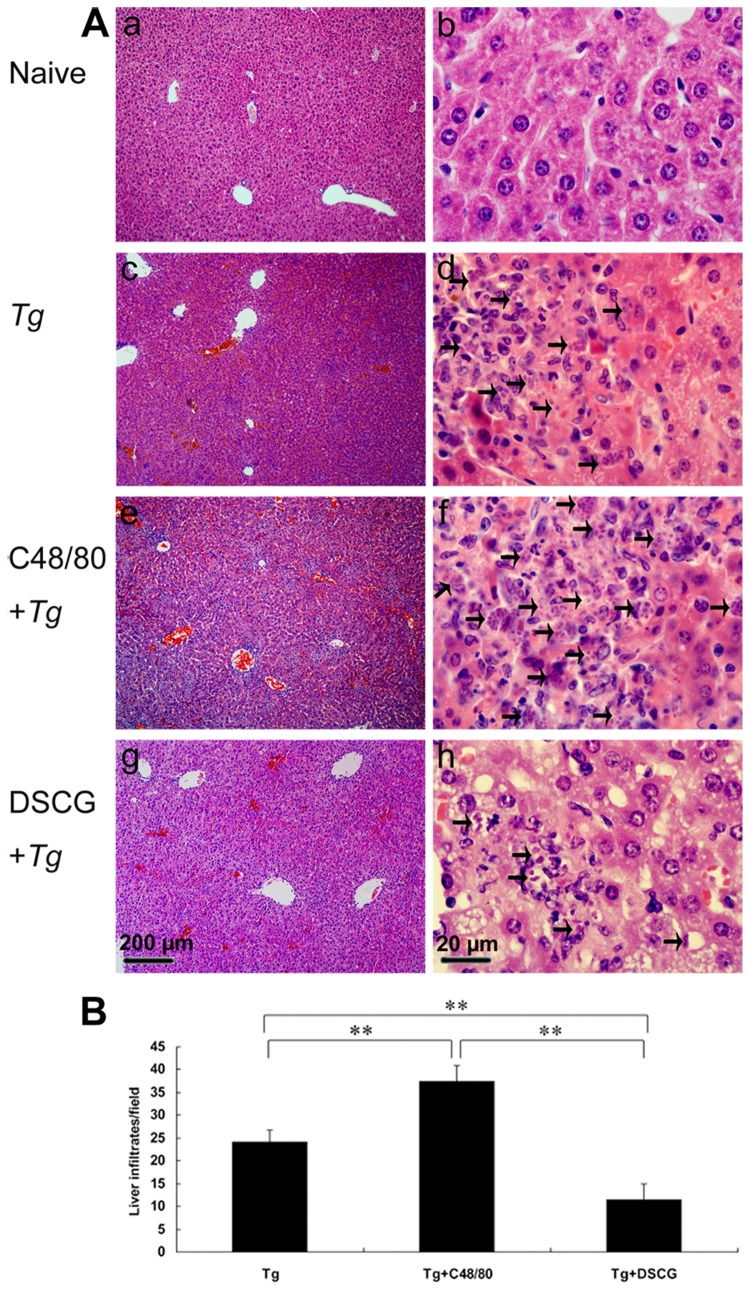
The liver histological analysis of *T*. ***gondii*-infected mice from different groups**. Infected mice i.p. inoculated with 10^2^ RH tachyzoites of *T. gondii* were killed at 9-10 days p.i. (A) Representative microscopic pictures show sections from uninfected mouse treated with PBS (a and b), infected control mouse (c and d), infected mouse treated with C48/80 (e and f), and infected mouse treated with DSCG (g and h). Tachyzoites were indicated with arrows. H&E stain. (B) Quantitative analysis of the number of inflammatory foci per field in liver sections from different groups. There were 4 mice per group, and the data are representative of two experiments. *, *P* < 0.05; **, *P* < 0.01 (compared to control).

**Figure 8:**
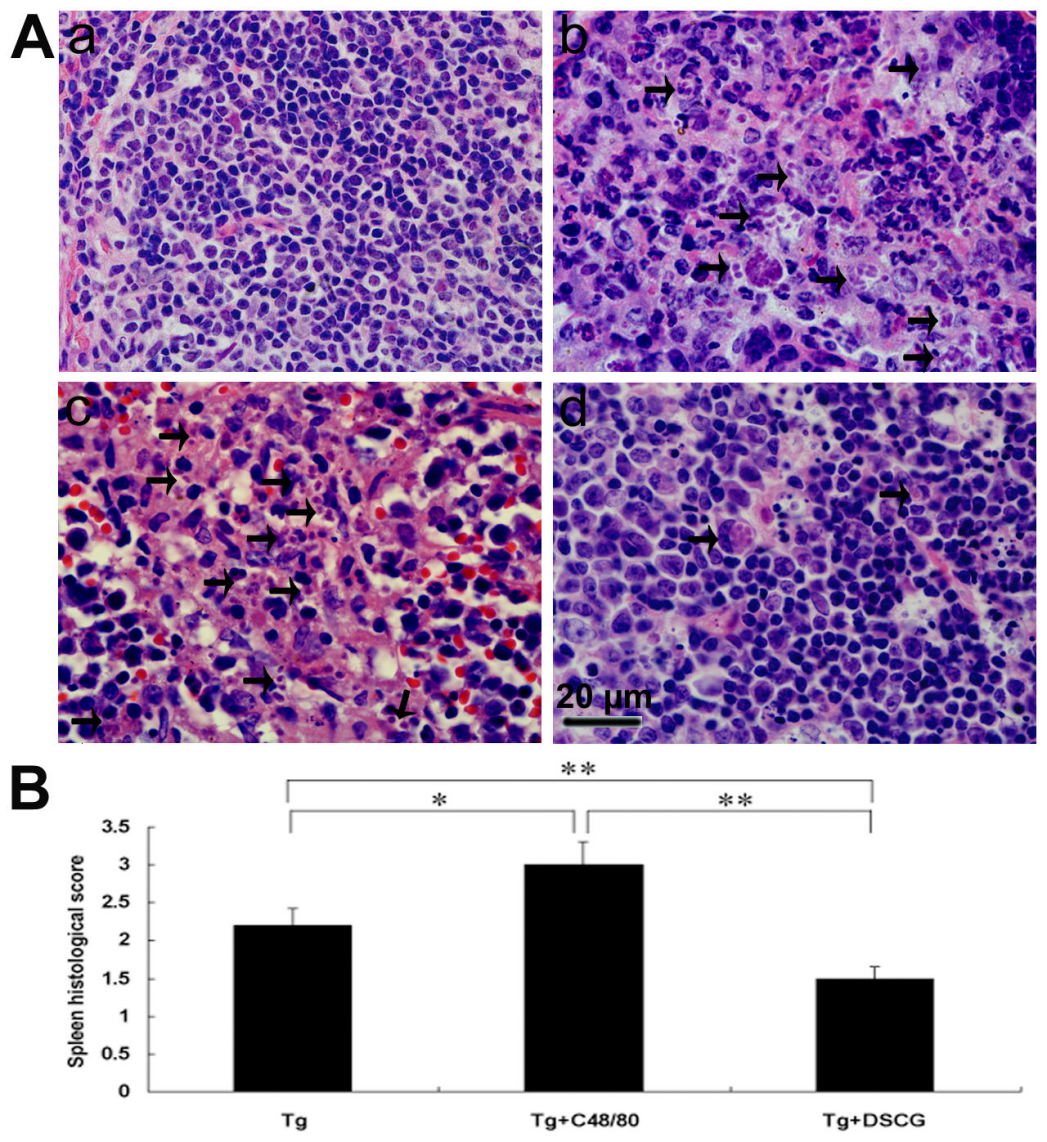
The spleen histological analysis of *T*. ***gondii*-infected mice from different groups**. Infected mice i.p. inoculated with 10^2^ RH tachyzoites of *T. gondii* were killed at 9-10 days p.i. (A) Representative microscopic pictures show sections from uninfected mouse treated with PBS (a), *T. gondii*-infected control mouse (b), *T. gondii*-infected mouse treated with C48/80 (c), and *T. gondii*-mouse treated with DSCG (d). Tachyzoites were indicated with arrows. H&E stain. (B) Histological score analysis of spleen tissues. There were 4 mice per group, and the data are representative of two experiments. *, *P* < 0.05; **, *P* < 0.01 (compared to control).

**Figure 9:**
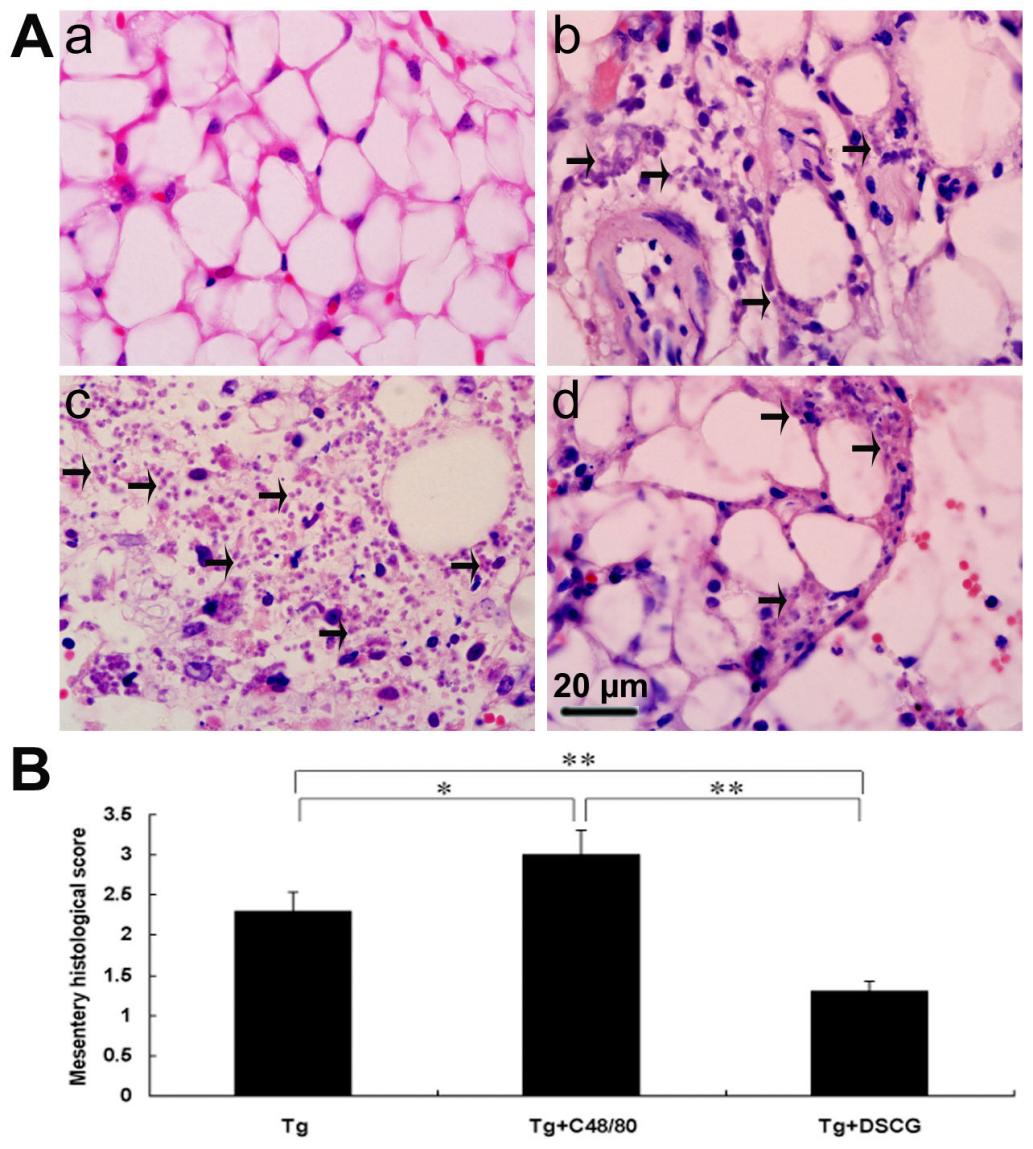
The mesentery histopathology of *T*. ***gondii*-infected mice from different groups**. Infected mice i.p. inoculated with 10^2^ RH tachyzoites of *T. gondii* were killed at 9-10 days p.i. (A) Representative microscopic pictures show sections from uninfected mouse treated with PBS (a), *T. gondii*-infected control mouse (b), *T. gondii*-infected mouse treated with C48/80 (c), and *T. gondii*-infected mouse treated with DSCG (d). Tachyzoites were indicated with arrows. H&E stain. (B) Histological score analysis of mesentery tissues. There were 4 mice per group, and the data are representative of two experiments. *, *P* < 0.05; **, *P* < 0.01 (compared to control).

To investigate the effects of the mediators released by MCs on tissue pathological changes, the liver (Figure 7A), spleen (Figure 8A), and mesentery (Figure 9A) tissues from different groups were examined histological. Control sections of liver (Figures 7a and b), spleen (Figure 8a), and mesentery (Figure 9a) from uninfected mice treated with PBS were negative for both inflammation and necrosis foci and *T. gondii* staining. After primary i.p. *T. gondii* RH strain infection, severe damage (obvious inflammation and necrosis foci) and a great number of RH tachyzoites were observed in the liver (Figure 7c and d), spleen (Figure 8b), and mesentery (Figure 9b) tissues of infected control mice. In comparison, even severer damage (stronger inflammation and more necrosis foci) and a greater number of RH tachyzoites were observed in the liver (Figure 7e and f), spleen (Figure 8c), and mesentery (Figure 9c) tissues of *T. gondii*-infected mice treated with C48/80; whereas attenuated or moderate histological evidence (mild inflammation and fewer necrosis foci) and a lower number of RH tachyzoites were observed in the liver (Figure 7g and h), spleen (Figure 8d) and mesentery (Figure 9 d) tissues of *T. gondii*-infected mice treated with DSCG. Treatment with C48/80 or DSCG did not change the tissue histology from uninfected mice, comparing with that of uninfected mice received PBS (data not shown). 

Quantitative analysis of the severity of inflammation and necrosis of liver sections (e.g. the number of inflammatory foci per field, 3 slides/animal) of different groups of mice was performed (Figure 7B). A great number of inflammatory foci of neutrophil infiltrates were observed in the liver of *T. gondii*-infected control mice. In comparison, significantly increased inflammatory foci of neutrophil infiltrates were observed in the *T. gondii*-infected mice with C48/80 treatment (*P* < 0.01), whereas significantly reduced inflammatory foci of neutrophil infiltrates were observed in the *T. gondii*-infected mice with DSCG treatment (*P* < 0.01). Semiquantitative histological evaluation of spleen (Figure 8B) and mesentery (Figure 9B) sections (3 slides/animal) of different groups of mice were performed. Severe pathology was shown in the spleen and mesentery tissues of *T. gondii*-infected mice without treatment. In comparison, even severer pathology were shown in the spleen and mesentery tissues of *T. gondii*-infected mice with C48/80 treatment (*P* < 0.05); whereas attenuated pathology were shown in the spleen and mesentery tissues of infected mice with DSCG treatment (*P* < 0.01). 

### Increased parasite burden in *T. gondii*-infected mice with C48/80 treatment

**Figure 10:**
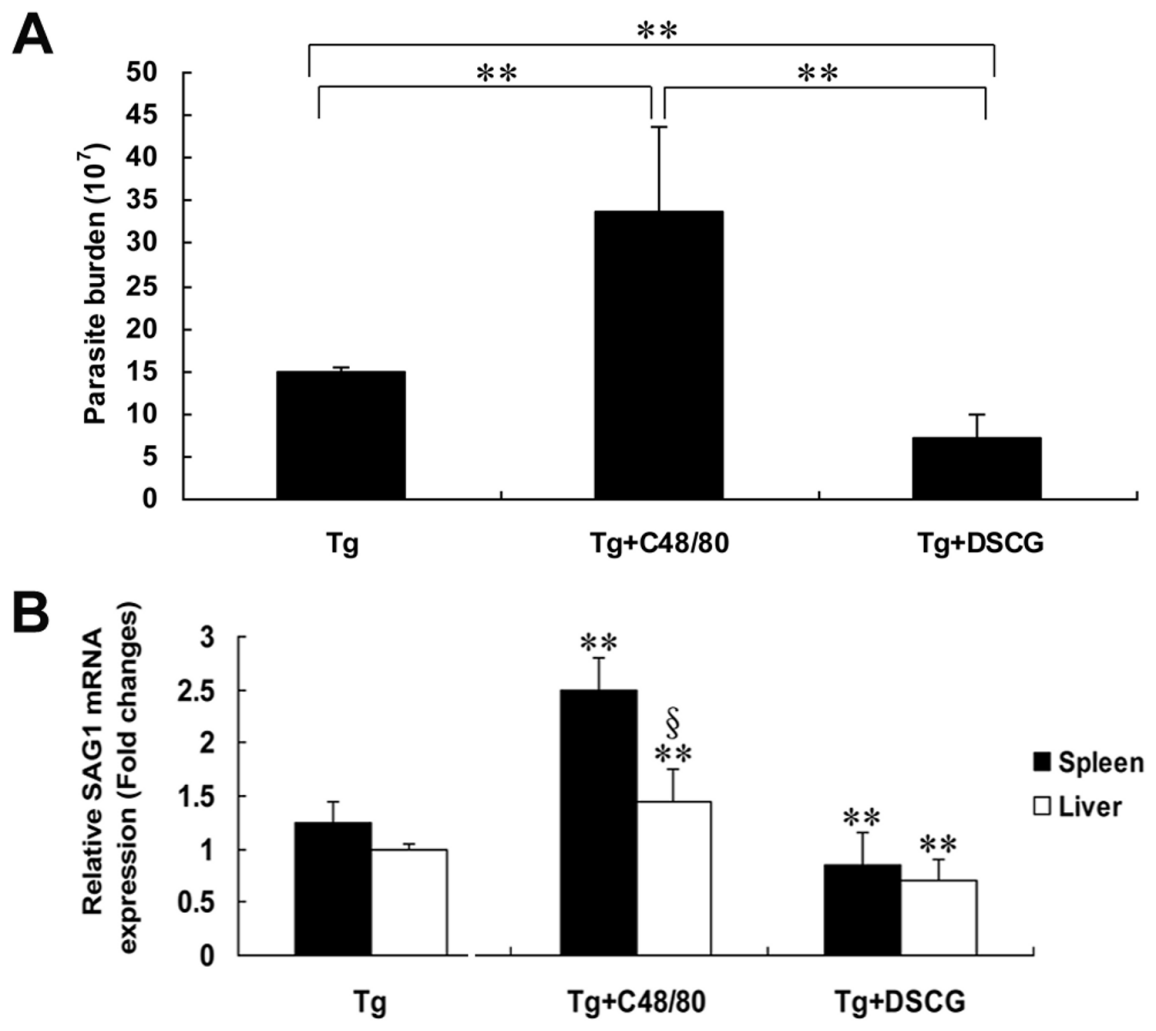
Parasite burden of *T*. ***gondii* RH strain tachyzoites in the peritoneal lavage fluids and tissues**. (A) Parasite burden of *T. gondii* RH strain tachyzoites in the peritoneal lavage fluids and (B) normalized mRNA expression levels of *T. gondii* tachyzoite SAG1 gene in the spleens and livers using qRT-PCR, from different groups i.p. inoculated with 10^2^ *T. gondii* RH strain tachyzoites at 9-10 days p.i. There were 4 mice per group, and the data are representative of two experiments. Symbols indicate statistically significant differences (*P* < 0.01) for comparison with the uninfected controls (**) and for comparison between group means (§).

To investigate whether MC activation and degranulation are important in host defense, live *T. gondii* tachyzoites were recovered from the peritoneal lavage fluids of infected mice with either C48/80 or DSCG treatment, or without treatment at 9-10 days p.i when mice were becoming moribund, and counted by hemocytometer (Figure 10A). Compared with *T. gondii*-infected control mice, there was a significant increase (2.3-fold) in the number of *T. gondii* tachyzoites in the peritoneal lavage fluids of infected mice treated with C48/80 (*P* < 0.01), whereas there was a significant decrease (2.1-fold) in the number of *T. gondii* tachyzoites in that of mice treated with DSCG (*P* < 0.01). In addition, a significant decrease (4.8-fold) in the number of *T. gondii* tachyzoites from infected mice treated with DSCG in comparison with that from infected mice treated with C48/80 (*P* < 0.01). To confirm the parasite burden of *T. gondii* tachyzoite in tissues, qRT-PCR was performed to determine the levels of mRNA transcripts for tachyzoite SAG1-stage specific gene in both liver and spleen tissues from different groups of mice at 9-10 days p.i (Figure 10B). Compared with *T. gondii*-infected controls, there was a significantly increased mRNA transcripts for SAG1 in both liver (*P* < 0.01) and spleen (*P* < 0.01) of infected mice treated with C48/80, whereas there was a significantly decreased mRNA transcripts for SAG1 in both liver (*P* < 0.01) and spleen (*P* < 0.01) of infected mice treated with DSCG (*P* < 0.01). 

### Th1 and Th2 mRNA cytokine responses in the spleen and liver of different groups

**Figure 11:**
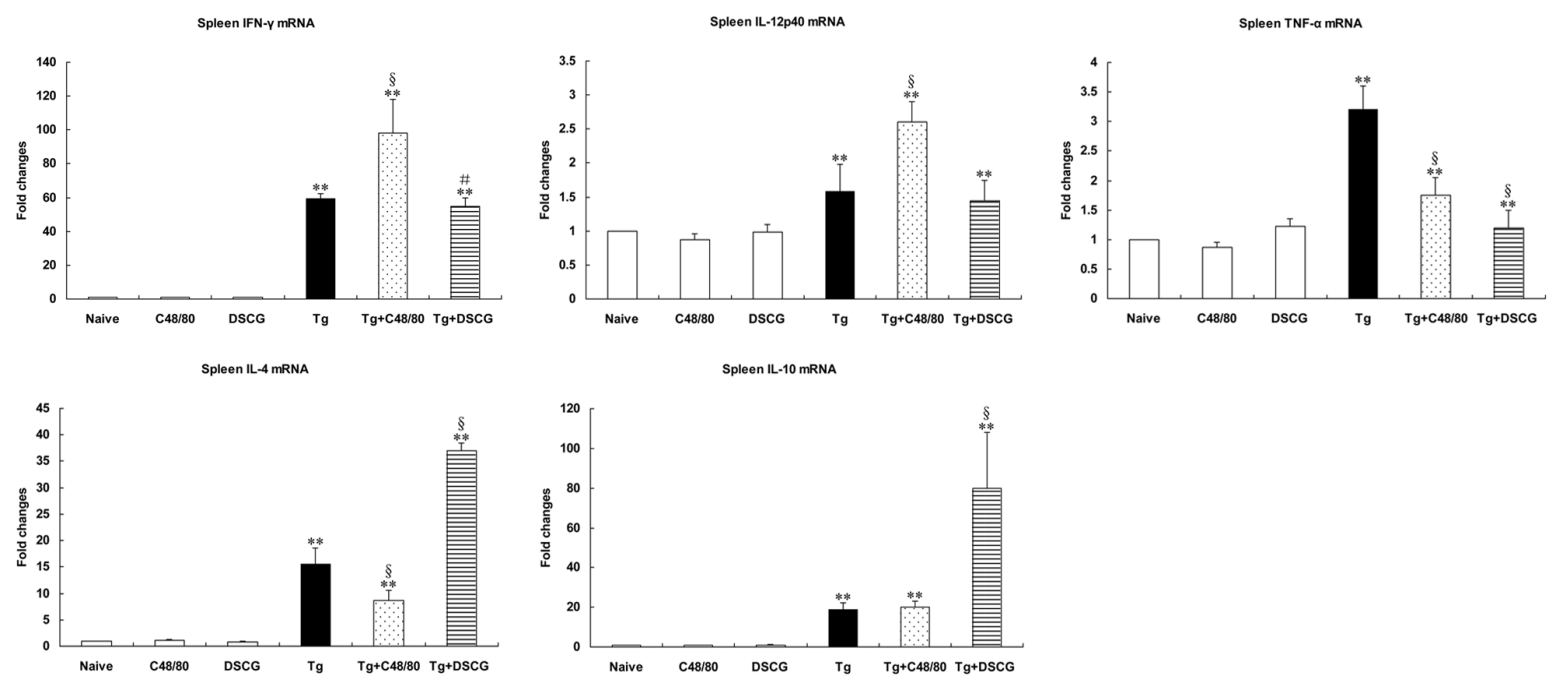
Cytokine mRNA expressions in spleens from different groups i.p inoculated with 10^2^ *T*. ***gondii* RH strain tachyzoites at 9-10 days p.i., using qRT-PCR**. There were 4 mice per group, and the data are representative of two experiments. Symbols indicate statistically significant differences (*P* < 0.01) for comparison with the uninfected control mice (**) and the infected controls (§), and statistically significant differences (*P* < 0.05) for comparison with the infected controls (#).

**Figure 12:**
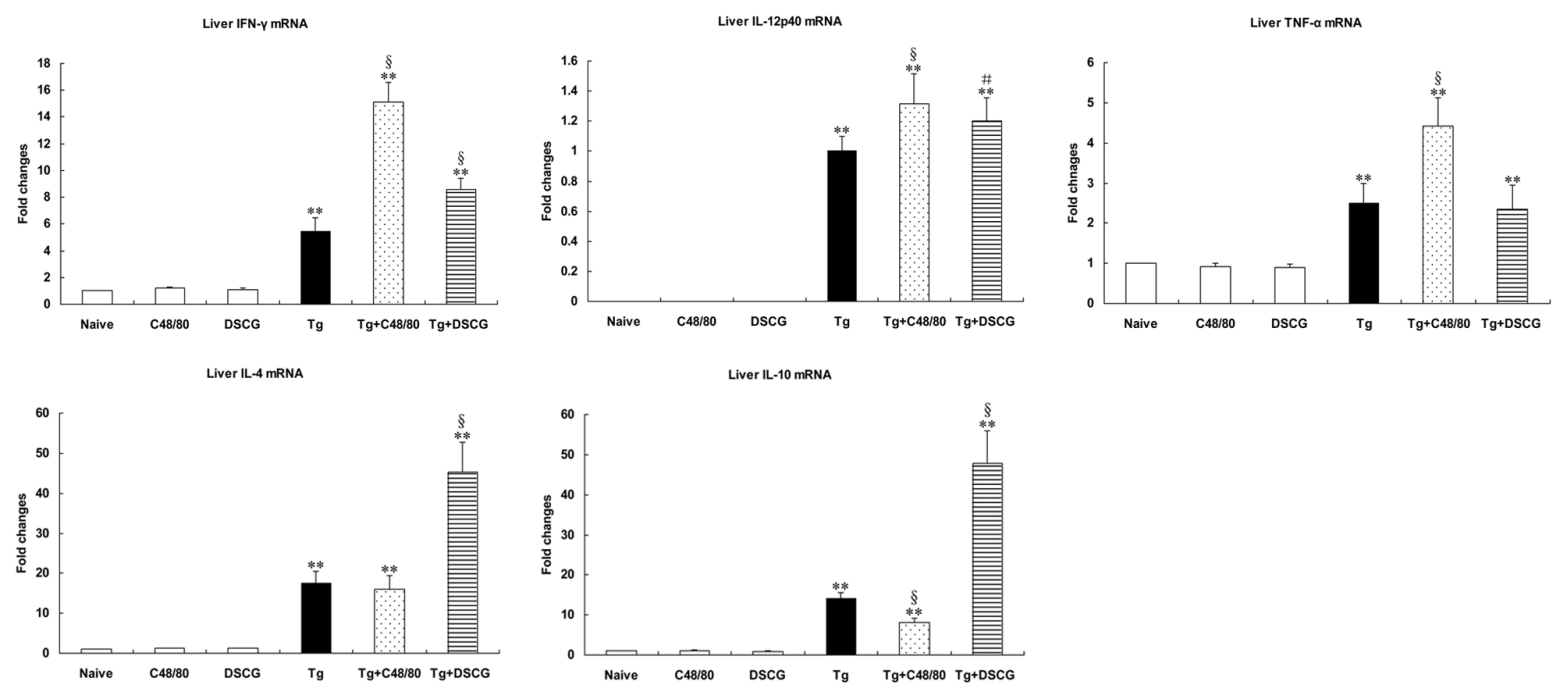
Cytokine mRNA expressions in livers from different groups i.p. inoculated with 10^2^ *T*. ***gondii* RH strain tachyzoites at 9-10 days p.i., using qRT-PCR**. There were 4 mice per group, and the data are representative of two experiments. Symbols indicate statistically significant differences (*P* < 0.01) for comparison with the uninfected control mice (**) and the infected controls (§), and statistically significant differences (*P* < 0.05) for comparison with the infected controls (#).

The effect of MC mediator release on Th1 and Th2 cytokine responses after *T. gondii* infection was evaluated by measuring IFN-γ, IL-12p40, TNF-α, IL-4, and IL-10 mRNA expressions in the spleens (Figure 11) and livers (Figure 12) of different groups. Cytokine mRNA expressions in naïve mice were not altered by C48/80 or DSCG treatment itself. However, compared with uninfected mice treated with PBS, there were significantly increased mRNA expressions of IFN-γ, IL-12p40, TNF-α, IL-4, and IL-10 in the livers and spleens of *T. gondii*-infected control mice at days 9-10 p.i. (*P* < 0.01), using qRT-PCR. Compared with *T. gondii*-infected controls, the Th1 cytokine (IFN-γ, IL-12p40, and TNF-α) expressions were significantly increased (*P* < 0.01) and the Th2 cytokine (IL-10) was significantly decreased (*P* < 0.01) in the livers, and the expressions of IFN-γ (*P* < 0.01) and IL-12p40 (*P* < 0.01) were significantly increased but TNF-α (*P* < 0.01) and IL-4 (*P* < 0.01) were significantly decreased in the spleens of infected mice treated with C48/80 at day 9-10 p.i. Whereas the expressions of Th1 cytokine [IFN-γ (*P* < 0.01) and IL-12p40 (*P* < 0.05)] were significantly increased in the liver, and IFN-γ (*P* < 0.05) and TNF-α (*P* < 0.01) were significantly decreased in the spleens of the infected mice treated with DSCG at day 9-10 p.i. However, the Th2 cytokine (IL-4 and IL-10) expressions in both spleen and liver were significantly increased (*P* < 0.01) in the infected mice treated with DSCG. 

## Discussion

Toxoplasmosis, an inflammatory disease, can lead to severe pathology in both humans and animals, involves leukocyte recruitment, immune responses, and inflammatory cytokine production, and immunity to infection relies on the development of a strong cell-mediated immune response, such as T cells and the production of IFN-γ [17,18]. It has been reported that a significant increase in the number of MCs in animals inoculated either i.p. or via the conjunctiva with *T. gondii* tachyzoites has been observed previously [19]. After incubation with *T. gondii* in vitro, peritoneal lavage MCs of Sprague-Dawley rats were found to degranulate and release LTB4, which damage *T. gondii* tachyzoites [20]. Despite these observations, little is known about the relationship between MCs and pathogenesis of *T. gondii* infection, as well as the MCs mediators generated in response to *T. gondii* infection. KM mice are the most widely used outbred colony in China. Using this murine model of *T. gondii* infection in the present study, we show here that the activation of MCs in mice via C48/80 injection resulted in increased *T. gondii* load and enhanced inflammatory infiltrate, whereas the MC stabilization with DSCG diminished *T. gondii* load and attenuated inflammatory reaction. Our data demonstrate that MCs play a crucial role in the course of *T. gondii* infection. 

In this study, our data demonstrated that infection with *T. gondii* not only increased the number of MCs in the analyzed tissues but also induced noticeable MC degranulation at 9-10 days p.i., by both toluidine blue staining and immunofluorescence staining of tryptase. As it has been reported that immunohistochemical staining for tryptase is a highly specific and sensitive method for identifying MCs [21], we also found MC density was significantly higher with immunofluorescence staining of tryptase compared with that of toluidine blue staining, due to the strong immunofluorescence staining of both intact and degranulated MCs. MC activation and degranulation most commonly result from multivalent antigens binding to the IgE bound to the high-affinity IgE receptor (FcεRI) on the surface, which results in noncytotoxic degranulation and the release of a variety of preformed and newly synthesized mediators [22]. The degranulation of MCs observed in *T. gondii*-infected animals is probably due to the presence of excreted-secreted antigens from *T. gondii* in tissues [23]. The C48/80 has been used to study allergies and anaphylaxis, because it can vigorously activate the release of histamine via the mechanism of cellular exocytosis [24]. In vivo studies have shown that C48/80 is a potent activator of MCs [25], a receptor mimetic that directly activates G proteins and stimulates vigorous MC degranulation, and releasing MC mediators independently of FcεRI activation [26]. Thus, C48/80 has been widely used to degranulate MCs in live animals. To determine whether regulation of MC activation controls acute toxoplasmosis, we injected C48/80 into *T. gondii*-infected mice before infection with *T. gondii*, and mice received daily injection of C48/80 during the experiment. Thus, MCs are repeatedly stimulated to release mediators under the conditions used in the present study. Compared with infected controls, in *T. gondii*-infected mice with C48/80 treatment, the presence of normal numbers of degranulated MCs containing granules at the site of infection with *T. gondii* correlates with the development of severer pathology, which presented as significantly more inflammation sites or higher pathological scores. Pharmacological treatment of mice with C48/80 triggers MC activation and the release of preformed mediators such as histamine, tryptase, chemokines, and interleukins that are important in the initial events of the inflammatory response [27]. DSCG is a drug widely used in the treatment of asthmatic patients [28], and observations from in vitro tests and animal models show that the effect of DSCG is related to MC stabilization [14]. DSCG prevents MC degranulation and acts as antiinflammatory agent [29], and the effect of DSCG is due to its ability to stabilize the MC membrane and to prevent release of histamine and inflammatory mediators. In the current study, compared with infected controls, there were significantly increased MC numbers in the spleens, accompanied with significantly impaired pathogenesis of *T. gondii* infection in the analyzed tissues of the infected mice with DSCG treatment. Our data suggest that mediators released by MCs results in impairment of *T. gondii* clearance and reduced MC degranulation limits pathogenesis caused by *T. gondii* infection, which indicates that MC activation/inhibition mechanisms are potential novel targets for *T. gondii* infection prevention and control. 

It is well known that activated MCs synthesize and release a large number of cytokines and chemokines [30]. To directly evaluate the in vivo role of MCs in acute murine toxoplasmosis, the effect of MC mediator release on Th1 and Th2 cytokine responses was evaluated in the spleens and livers in different groups. Importantly, increased pathogenesis of *T. gondii* infection, accompanied with enhanced mRNA expressions of Th1 cytokine (IFN-γ, IL-12p40, or TNF-α), and decreased Th2 cytokine (IL-4 or IL-10) in liver and spleen in C48/80-treated mice, suggesting that C48/80 promotes MC activation or degranulation and thereby affects the release of MC mediators. MC degranulation produces the initial signals responsible for regulating neutrophil and mononuclear cell recruitment in the bronchoalveolar space through release of both pro- and antiinflammatory mediators [27]. Activation of MCs and the subsequent release of their granular constituents is a major mechanism whereby MCs participate in pathobiological processes [31]. These findings suggest that release of mediators after MC activation plays an important role in modulating acute inflammation during *T. gondii* infection. MCs likely affect pathogenesis of *T. gondii* infection by up-regulating the expressions of Th1 cytokine (IFN-γ, IL-12p40, or TNF-α), and down-regulating the expressions of Th2 cytokine (IL-4 or IL-10), but other unmeasured mediators may also involve this process. Whereas infected mice treated with DSCG, the expressions of Th1 cytokine (IFN-γ or TNF-α) were significantly decreased and Th2 cytokine (IL-4 and IL-10) were significantly increased in the spleens or livers. IL-4 is the main promoter of type-2 responses and is classically reported as counter-regulating type-1 immunity [32], and IL-10 plays a vital role in controlling the inflammatory response during acute *T. gondii* infection [33]. In the course of toxoplasmosis in patients, the level of IL-10 is five-fold higher than that in healthy controls; however, the levels of IL-12 and TNF-α are comparable to those observed in healthy controls [34]. MCs and MC-derived IL-10 limit leukocyte infiltration, inflammation, and tissue damage associated with immunological or innate responses [9]. Histamine, the main preformed mediator stored in MC granules, stimulates alveolar macrophages to release neutrophil and monocyte chemotactic factors [27]. In the present study, the role played by MCs in neutrophil recruitment in analyzed tissues may be attributed to the consequence of a reduction in the expressions of IL-4 or IL-10 at the site of the infection in infected mice with C48/80 treatment. In addition, the results presented here may also be due to an indirect effect of the release of mediators by MCs on the production and release of cytokines and chemokines by other cells. MCs have been proposed to be an important source of TNF [35]. However, MCs do not contribute to the rapid appearance of TNF in the serum of LPS-treated mice [36]. Our data showed that MCs contribute significantly to local (the liver tissue) TNF-α production in this experimental model. 

IFN-γ-mediated immune responses are essential for controlling tachyzoite proliferation during both acute acquired infection and reactivation of infection in the brain [37], and IFN-γ has also been demonstrated to regulate the *T. gondii* load and interconversion in the eye [38]. However, there were increased IFN-γ mRNA expressions in both livers and spleens in mice treated with C48/80 in this study, thus, the inability to control *T. gondii* replication observed in mice treated with C48/80 seems not to be a consequence of an increase in the expression of IFN-γ. IL-4 is protective against development of TE by preventing formation of *T. gondii* cysts and proliferation of tachyzoites in the brain [39]. In this study, there were significantly decreased levels of IL-4 and IL-10 in spleen and liver, respectively, from mice treated with C48/80. It has been reported that IL-10 limits parasite burden in murine *Trypanosoma cruzi* infection [40], and IL-10 mRNA levels directly correlate with parasite load in lesions tissues of post kala azar dermal leishmaniasis patients [41]. This finding suggests that mediators released by C48/80-treated MCs result in impairment of *T. gondii* clearance, which may be related to the decreased IL-4 or IL-10 levels; whereas infected mice treated with DSCG result in lower parasite burden, which may be related to the increased IL-4 and IL-10 levels in this model. Our data indicated that MC activation is important in the regulation of the inflammatory response to host defense against *T. gondii* infection, and the cellular immune response may be partially impaired in infected mice treated with C48/80, which is crucial to the destruction and elimination of *T. gondii*. We cannot outline the mechanism increasing the parasite burden in acute toxoplasmosis with C48/80 treatment in the current study; however, the fact that it involves MCs degranulation brings new aspect of the problem. In addition, we found that the levels of *T. gondii* -specific IgG were no differences among the infected groups (data not shown), which suggested that the administration of either C48/80 or DSCG does not change the humoral immunity during acute *T. gondii* infection. 

In summary, this study showed that MC stimulator were able to deteriorate the pathology and increase parasite burden in *T. gondii*-infected mice with C48/80 treatment; whereas MC stabilizers were able to improve the pathology and decrease parasite burden in *T. gondii*-infected mice with DSCG treatment. Our data indicate that MCs contribute to susceptibility and systemic inflammation during acute murine *T. gondii* infection through the production and secretion of mediators including cytokines that play a role in the recruitment and activation of inflammatory cells in this experimental model, and these findings propose a novel mechanism that MCs play important roles for host immunity against *T. gondii* infection. 
